# NH_3_-Induced In Situ Etching Strategy Derived 3D-Interconnected Porous MXene/Carbon Dots Films for High Performance Flexible Supercapacitors

**DOI:** 10.1007/s40820-023-01204-4

**Published:** 2023-10-18

**Authors:** Yongbin Wang, Ningjun Chen, Bin Zhou, Xuefeng Zhou, Ben Pu, Jia Bai, Qi Tang, Yan Liu, Weiqing Yang

**Affiliations:** 1 Key Laboratory of Advanced Technologies of Materials, Ministry of Education, School of Materials Science and Engineering, Southwest Jiaotong University, Chengdu, 610031 People’s Republic of China; 2 Research Institute of Frontier Science, Southwest Jiaotong University, Chengdu, 610031 People’s Republic of China; 3 Sichuan Research Center of New Materials, Institute of Chemical Materials, China Academy of Engineering Physics, Chengdu, 610200 People’s Republic of China

**Keywords:** Ti_3_CNT_*x*_ MXene, Carbon dots, In situ etching, 3D-interconnected porous structure, Flexible supercapacitors

## Abstract

2D MXene (Ti_3_CNT_*x*_) has been considered as the most promising electrode material for flexible supercapacitors owing to its metallic conductivity, ultra-high capacitance, and excellent flexibility. However, it suffers from a severe restacking problem during the electrode fabrication process, limiting the ion transport kinetics and the accessibility of ions in the electrodes, especially in the direction normal to the electrode surface. Herein, we report a NH_3_-induced in situ etching strategy to fabricate 3D-interconnected porous MXene/carbon dots (p-MC) films for high-performance flexible supercapacitor. The pre-intercalated carbon dots (CDs) first prevent the restacking of MXene to expose more inner electrochemical active sites. The partially decomposed CDs generate NH_3_ for in situ etching of MXene nanosheets toward 3D-interconnected p-MC films. Benefiting from the structural merits and the 3D-interconnected ionic transmission channels, p-MC film electrodes achieve excellent gravimetric capacitance (688.9 F g^−1^ at 2 A g^−1^) and superior rate capability. Moreover, the optimized p-MC electrode is assembled into an asymmetric solid-state flexible supercapacitor with high energy density and superior cycling stability, demonstrating the great promise of p-MC electrode for practical applications.

**Supplementary Information:**

The online version contains supplementary material available at 10.1007/s40820-023-01204-4.

## Highlights

- Porous MXene (p-MC) films with 3D-interconnected ion transmission paths are constructed by NH_3_-induced in situ etching strategy.
- The flexible p-MC film electrodes exhibit a superior capacitance of 688.9 F g^−1^ with a good rate capability.
- The as-assembled p-MC solid-state flexible supercapacitors reveal superior electrochemical performance (99.7 F g^−1^ at 1 A g^−1^) and excellent flexibility.

**Supplementary Information:**

The online version contains supplementary material available at 10.1007/s40820-023-01204-4.

## Graphical abstract

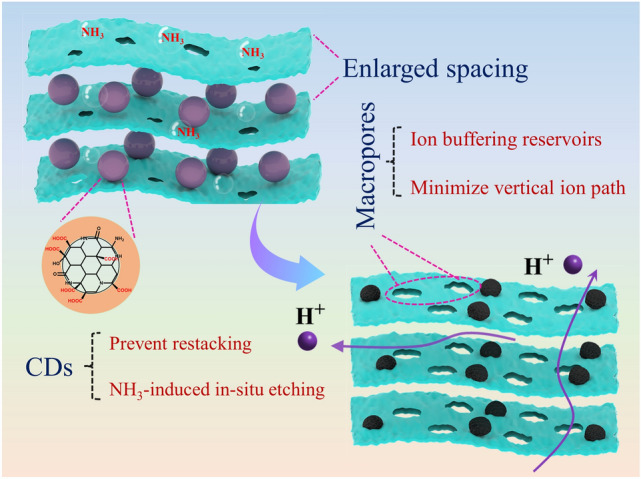

## Introduction

The past decades have witnessed the rapid development of portable and flexible electronics such as bendable screens [1, 2], electronic skins [3–6], flexible transistors [7], and wearable sensor devices [8–11]. Most of these flexible electronics require flexible power sources such as flexible supercapacitors [12–15]. In this regard, 2D MXenes are rising as ideal electrode materials for flexible and wearable supercapacitors due to their metallic conductivity, tunable chemical properties, unique flexibility, and excellent mechanical properties [16–21]. Moreover, MXene can store large number of charges through surface Faraday redox reaction, delivering much higher capacitance than traditional electric double-layer capacitors [22, 23]. However, the 2D MXene sheets are prone to restacking during the electrode fabrication process, which limits ion transport in the electrode and results in sluggish kinetics and inferior capacitance [24, 25].

Constructing ion transport channels is an effective way to resolve the abovementioned bottle-neck problem of 2D MXenes. For example, introducing spacers into MXene layers can effectively enlarge the interlayer spacing of MXene sheets. In this regard, transition metal oxides (e.g., TiO_2_, SnO_2_, and Fe_3_O_4_) [26–28], transition metal dichalcogenides (e.g., MoS_2_, and SnS_2_,) [29, 30], polymers (e.g., polypyrrole, and polyaniline) [31, 32], and low-dimensional carbon materials [e.g., graphene, carbon nanotubes, and carbon dots (CDs)] [33–36] have been successfully inserted into MXene layers, thus creating substantial ion transport channels in the horizontal direction, and exposing more internal electrochemical active sites. However, the spacer-intercalation strategy cannot reduce the high ion-path tortuosity in the direction normal to the MXene films, which would be an obstacle for fast ion transport and high rate capability. On the other hand, constructing in-plane pores on MXene sheets can effectively reduce the ion-path tortuosity and generate ion-transport microchannels in the vertical direction [37–40]. However, this approach cannot increase the interlayer spacing of MXene sheets, and thus the ion diffusion in the horizontal direction is still limited. In this context, it is highly anticipated that the combination of introducing interlayer spacer and creating in-plane pores could form 3D interconnected ion transport channels and expose more internal electrochemical active sites, which would simultaneously promote the capacitance and rate capability for the electrode.

In this work, we propose a new strategy to construct a 3D-interconnected porous MXene/Carbon dots (p-MC) films with well-distributed in-plane macropores on MXene layers and tightly anchored CDs between MXene layers for high-performance flexible supercapacitors. In this novel structure, the in-plane macropores act as aortas to reduce the vertical ion-path tortuosity for fast ion diffusion and the enlarged interlayer spacing acts as capillaries to quickly transport the ions to each electrochemical active site. Benefiting from the structural advantages, the p-MC electrodes exhibit a high capacitance of 688.9 F g^−1^ at 2 A g^−1^, nearly 2.5 times that of the pure MXene. Also, the p-MC electrodes exhibit much higher pseudocapacitive contribution than that of pure MXene. When assembled into solid-state asymmetric flexible supercapacitors, the p-MC-based devices can exhibit an attractive electrochemical performance with a good flexibility.

## Experimental and Calculation

### Fabrication of Ti_3_CNT_***x***_ MXene Solution and CDs Aqueous Solution

The multilayered Ti_3_CNT_x_ MXene was prepared following our previous works [41]. Typically, 1 g of LiF (Chengdu Ke Long Co.) was added to 20 mL of 9 M HCl solution and stirred for 5 min. Then, 1.2 g of Ti_3_AlCN (400 mesh, purchased from 11 Technology Co., Ltd.) was added to the mixture and maintained at 40 °C for 24 h. The product was washed by deionized (DI) water repeatedly until the pH value of the supernatant reached 6. The precipitate was dispersed in 80 mL of DI water and sonicated for 1.5 h. After centrifugating at 3500 rpm for 60 min, the multilayered Ti_3_CNT_x_ colloidal solution was finally obtained.

The CDs were prepared by a reported solvothermal method [42, 43]. Specifically, 1 g of citric acid (CA) (Chengdu Ke Long Co.), 2 g of urea (Chengdu Ke Long Co.), and 10 mL of N, N-dimethylformamide (Chengdu Ke Long Co.) were mixed in a Teflon-lined autoclave and kept at 160 °C for 4 h. After cooling down to room temperature, the product was dialyzed in DI water for 3 days to remove the impurities and then centrifuged at 3500 rpm for 10 min to remove the oversized CDs.

### Preparation of p-MC and AC Electrodes

First, 3–18 mL of CDs solution (0.4 mg mL^−1^) was mixed with 10 mL of Ti_3_CNT_*x*_ (5.1 mg mL^−1^) solution and stirred for 30 min. Then, the mixture was filtered under vacuum through cellulose membrane and dried under vacuum at 30 °C to fabricate MXene/Carbon dots (MC) films. The p-MC electrodes were finally obtained by annealing the MC electrodes at 350 °C for 1 h at the heating rate of 10 °C min^−1^. The pure MXene films were prepared with the same method but without the addition of CDs.

As for the AC electrodes, 45 mg of AC (YP-50F, Kuraray) and 50 mg of polytetrafluoroethylene (PTFE, 10 wt% solution) were mixed and stirred for 10 min to form a suspension. Then, the suspension was dried at 80 °C to remove the solvent. The formed dough-like paste was repeatedly rolled into a thin AC film and cut into the same size (*d* = 12 mm) as p-MC electrodes.

### Construction of p-MC-Based Flexible Solid-State Supercapacitors

The gel electrolyte (PVA/H_2_SO_4_) was obtained by mixing the PVA (1799 type), deionized water, and concentrated H_2_SO_4_ (mass ration of 10:1:1) and stirring at 80 °C for 1 h [44]. To fabricate the p-MC-based supercapacitors, p-MC and AC films were pressed onto current collectors. Then, the PVA/H_2_SO_4_ electrolyte was uniformly coated on the surface of p-MC and AC electrodes, followed by pairing them face to face and pressing together. The flexible solid-state supercapacitors were finally obtained by packaging the assembled electrodes with polydimethylsiloxane (PDMS) and drying them at 30 °C until the PDMS completely cured [45].

### Material and Electrochemical Characterization

The morphologies of materials were characterized with the field emission scanning electron microscopy (SEM, FEI QUANTA FEG 250) and transmission electron microscopy (TEM, JEOL JEM-2100). The chemical structure of CDs was recorded by Fourier Transform Infrared Spectrometer (FTIR, TENSOR II). X-ray photoelectron spectroscopy (XPS) was recorded on Thermo Scientific ESCALAB 250Xi. Raman spectra were measured on confocal Raman microscope (HORIBA Jobin–Yvon XploRA).

The cyclic voltammetry (CV), galvanostatic charge–discharge (GCD), and electrochemical impedance spectroscopy (EIS) tests were performed on an electrochemical workstation (Chenhua CHI660E) in three-electrode system with AC electrode as counter electrode, Ag/AgCl in saturated KCl solution as reference electrode, and 1 M H_2_SO_4_ as electrolyte. The EIS tests were conducted in the frequency range of 100 kHz to 0.01 Hz at open circuit potential. The cycling tests of the p-MC-based flexible solid-state supercapacitor were carried out with a multi-channel galvanostat/potentiostat instrument (Arbin USA).

### Capacitance Calculations

The charge balance (Q^+^  = Q^−^) was achieved by balancing the mass of positive and negative electrodes according to the following equation:

1 $$\frac{m_+ }{{m_- }} = \frac{{C_{{\text{electrode}} - } \Delta V_- }}{{C_{{\text{electrode}} + } \Delta V_+ }}$$

where *m* is the loading mass of AC and p-MC electrode, *C*_electrode_ is the gravimetric capacitance, and *ΔV* is the potential window of each electrode.

The specific capacitance (*C*), power density (*P*), and energy density (*E*) were calculated according to the following equations:

2 $$C = \frac{\smallint IdV}{{2mv\Delta V}}$$

3 $$E = \frac{C\Delta V^2 }{{7200}}$$

4 $$P = \frac{3600vE}{{\Delta V}}$$

where *I* is the current, *v* is the scan rate.

## Results and Discussion

### Preparation of p-MC and Structural Characterizations

**Fig. 1:**
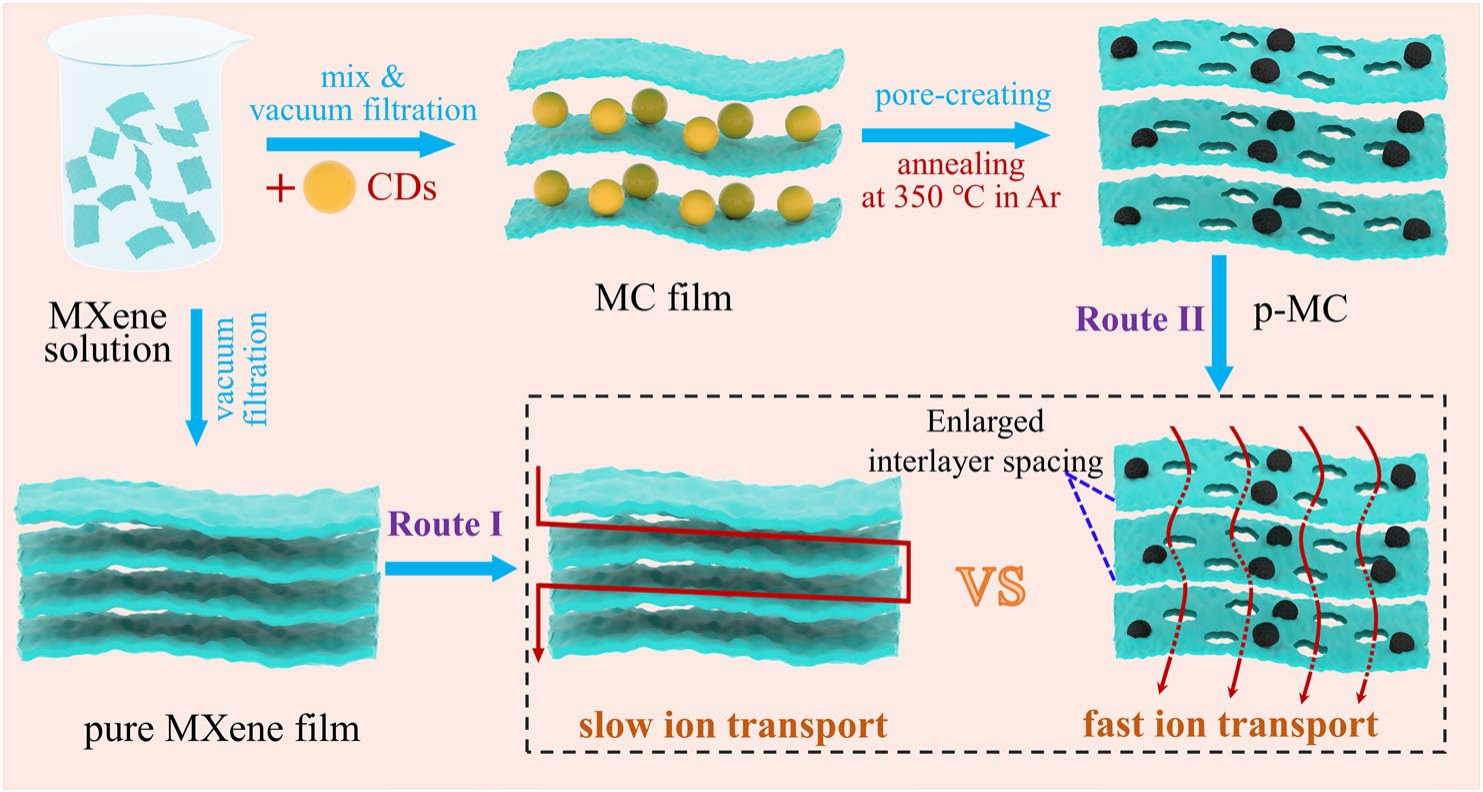
Schematic illustration of the fabrication process of p-MC film and the advantages of p-MC film over traditional pure MXene film

The design principle of p-MC composite films is schematically illustrated in Fig. 1. As shown in Route I, the conventional tightly stacked pure MXene films with a high ion-path tortuosity usually led to sluggish ion transport kinetics and low accessibility of electrochemical active sites to ions, which is detrimental to the rate performance and gravimetric capacitance of the electrodes. For comparison, the p-MC films obtained by vacuum filtration of a colloidal solution containing MXene nanosheets and CDs and annealing at 350 °C under Ar flow (shown in Route II) present three advantages: (i) the evenly intercalated CD spacers can effectively prevent the serious restacking of Ti_3_CNT_x_ nanosheets and broaden the interlayer spacing to expose more electrochemical active sites, thus increasing the horizontal ion-accommodation; (ii) the in-plane macropores can act as buffering reservoirs for electrolyte to reduce electrochemical response time; (iii) the in-plane porous structure can significantly reduce the ion-path tortuosity in the vertical direction, facilitating rapid ion transfer. These merits endow the p-MC electrodes with high capacitance and rate capability.

**Fig. 2:**
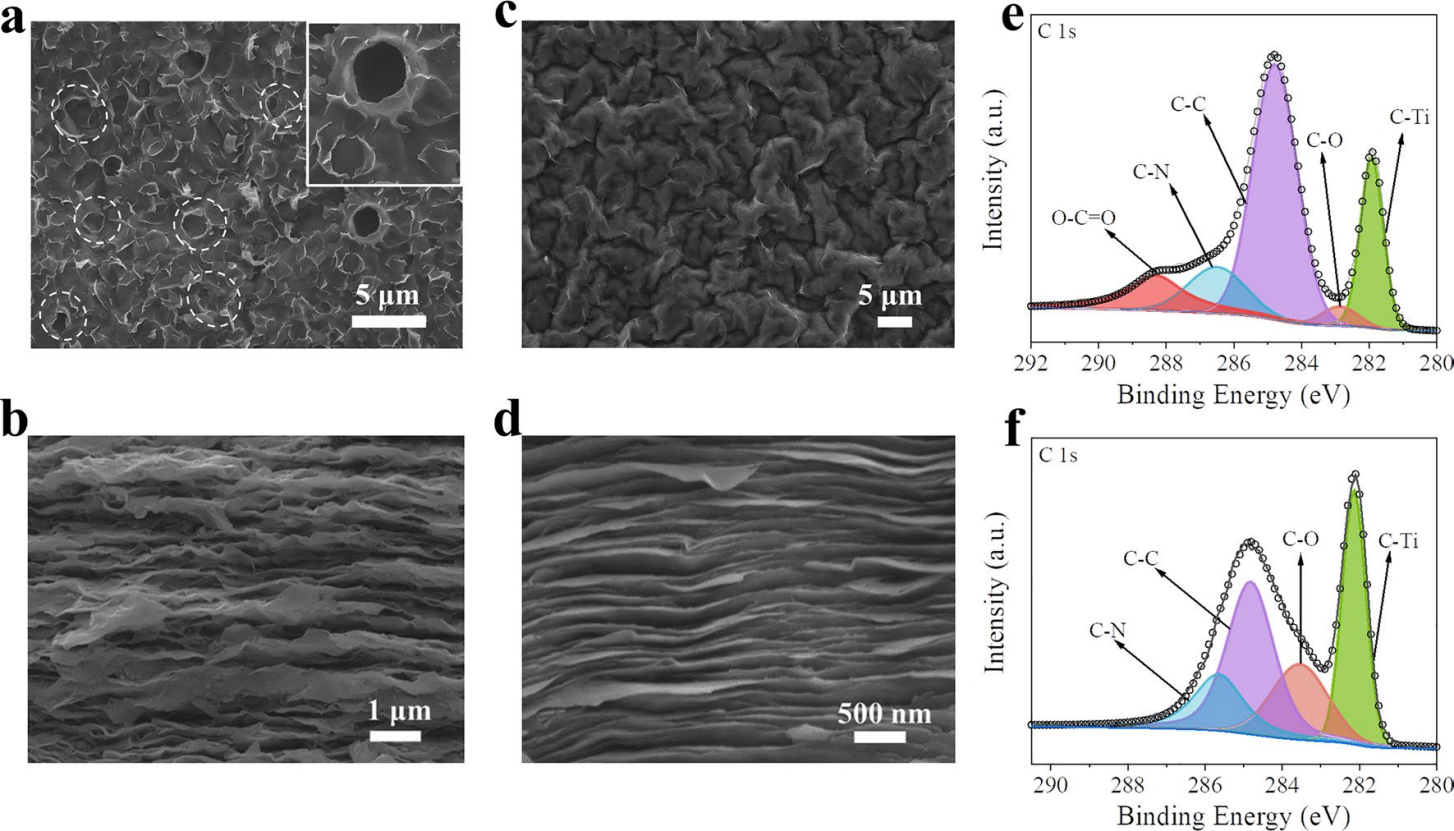
**a** Top-view and **b** cross-sectional SEM images of p-MC film. **c** Top-view and** d** cross-sectional SEM images for pure MXene film. The C 1*s *XPS spectra of **e** p-MC and **f** pure MXene films

As schematically illustrated in Fig. S1, the CDs were prepared through hydrothermal reactions (the detailed process can be found in Experimental Section). The TEM image in Fig. S2 shows that the obtained CDs possess uniform morphology with an average diameter of approximately 20 nm. Both CDs and MXene sheets are negatively charged in aqueous solutions (Fig. S3); thus, they can form a uniform and stable colloidal solution by mixing with each other owing to the electrostatic repulsion force. During the vacuum filtration process, the well dispersion of CDs and MXene sheets in the solution guarantees the uniform dispersion of CDs in the MXene/CDs (MC) composite film (the filter cake). In the thermal annealing process, the CDs with a variety of functional groups (e.g., −C=O, –COOH, –C–N) may react with the surface group (e.g., –OH, –F, and –O) on MXene sheets, thus anchoring the CDs onto the MXene sheets [46]. After thermal annealing, the MC film was converted to p-MC film. The SEM images show that the MXene layers in p-MC film are significantly wrinkled with expanded interlayer spacing and well-distributed in-plane macropores (Fig. 2a, b). Such a structure can effectively expose more internal electrochemical active sites and shorten the ion transport path, resulting in better electrochemical performance, especially for the gravimetric capacitance and rate performance. It should be noted that the flexibility of p-MC films can be still maintained during the constructing of porous structure (Fig. S4), showing a bright prospect in flexible devices. For comparison, the conventional pure MXene film shows a densely packed structure without in-plane pores (Fig. 2c, d), which is unfavorable for ion diffusion. From the Raman spectrum in Fig. S5, both pure MXene and p-MC exhibit similar Raman peaks located between ~ 100 and ~ 800 cm^−1^, which should be assigned to the* A*_*1g*_ and *E*_*g*_ group vibrations of Ti and C atoms and surface functional groups [47]. Comparing to the pure MXene, the two peaks of p-MC located at ~ 1350 and ~ 1580 cm^−1^ corresponding to the disordered-induced D-band and graphitic G-band of carbon, respectively, are much stronger, which should be ascribed to the contribution of CDs [47, 48]. X-ray diffraction (XRD) patterns indicate the intercalation of CDs does not change the basic crystal structure of p-MC (Fig. S6). XPS was carried out to identify the composition and chemical bonding information of pure MXene and p-MC films (Figs. 2e, f, and S8-S9). In the high-resolution C 1*s* XPS spectra (Fig. 2e, f), the p-MC shows a higher content of C–C bond than that of pure MXene. In addition, the peak at 288.3 eV, which corresponds to the –COOH group of CDs, can only be observed in p-MC [49–52]. These results confirm the successful insertion of CDs into MXene layers. Compare to the N 1*s* spectrums of pure MXene (Fig. S8a, b), the peaks at 398.7 and 400.1 eV correspond to the pyrrolic nitrogen and pyridinic nitrogen of CDs [53–55], respectively, can only be observed in p-MC films too. These results are further in accordance with the abovementioned inference.

### Formation Mechanism of 3D-Interconnected Pores for p-MC

**Fig. 3:**
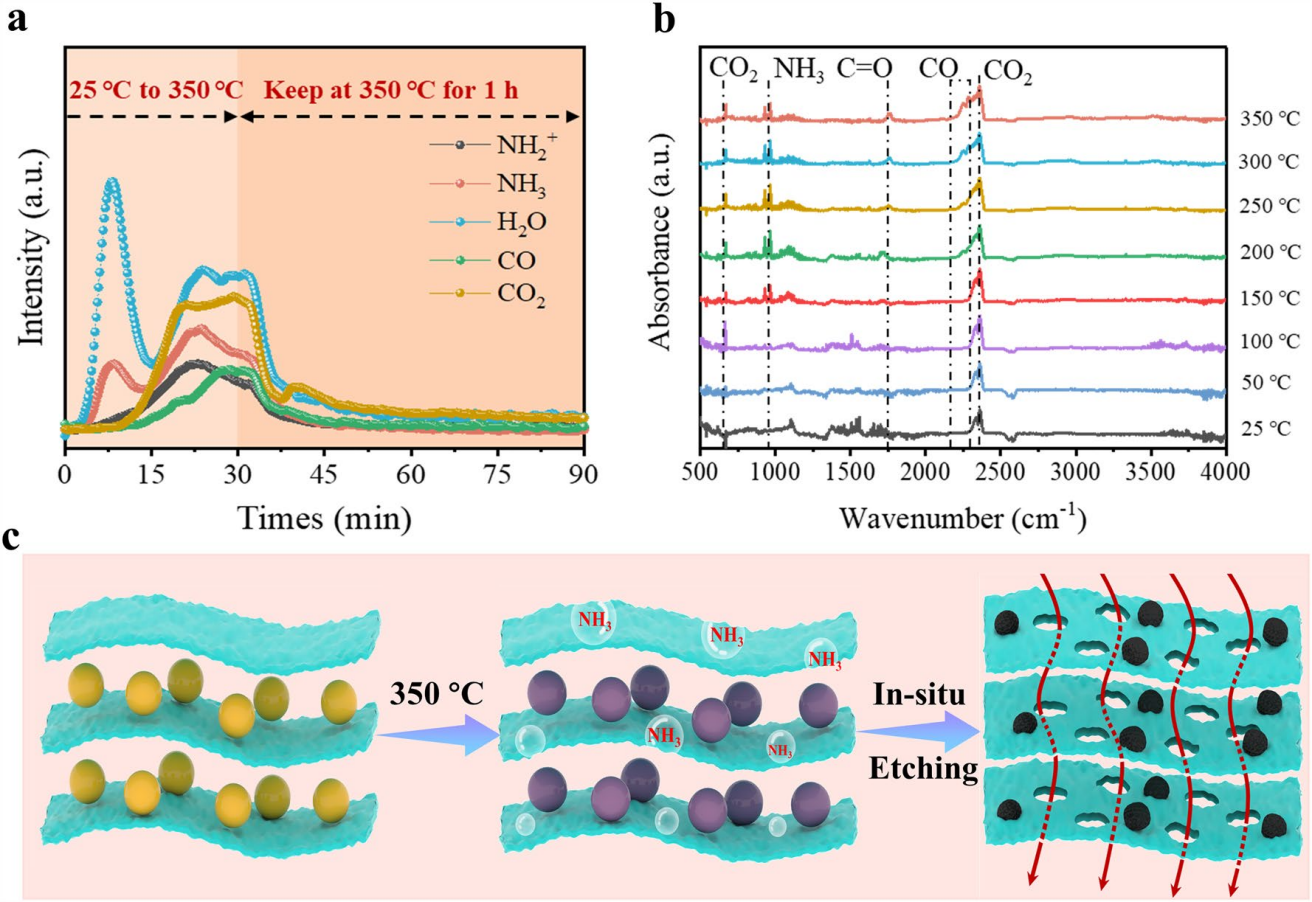
**a** Gaseous evolution curves of CDs during pyrolysis in TG-MS. **b** TG-FTIR spectra for volatiles at various temperatures for CDs. **c** Schematic diagram for the conversion process of p-MC film

To gain insight into the formation mechanism of p-MC film, a control experiment conducted under the identical synthetic conditions for p-MC film, but without CDs, produced a densely packed MXene film without any pores on its surface (Fig. S10), suggesting that the CDs play a critical role in the pore formation process. Considering no pores were observed in the pristine MC film before annealing (Fig. S11), the thermal annealing treatment is also a prerequisite for generating pores on MXene sheets. To further explore the pore formation mechanism of p-MC during the annealing process, in situ thermogravimetry-mass spectroscopy (TG–MS) and thermogravimetry-Fourier transform infrared spectroscopy (TG-FTIR) techniques were performed to monitor the gaseous products during the annealing of CDs. The TG-MS spectra (Fig. 3a) show that the pyrolysis of CDs generates many gaseous products, such as NH_3_, CO_2_, CO, and H_2_O [56–58]. The TG-FTIR spectra (Figs. 3b and S12) further confirm the generation of the abovementioned gases during the annealing process [59, 60]. To investigate whether these gases promote the formation of macropores on MXene sheets, we annealed pure MXene film under each of gases mentioned above and found that macropores were formed on the surface of MXene under NH_3_ atmosphere (Fig. S13). Based on the above results, we infer that the NH_3_ generated from the pyrolysis of CDs etches the MXene nanosheet during the annealing process, resulting in the formation of well-distributed in-plane macropores on MXene sheets (as illustrated in Fig. 3c).

### Electrochemical Performance in a Three-Electrode System

**Fig. 4:**
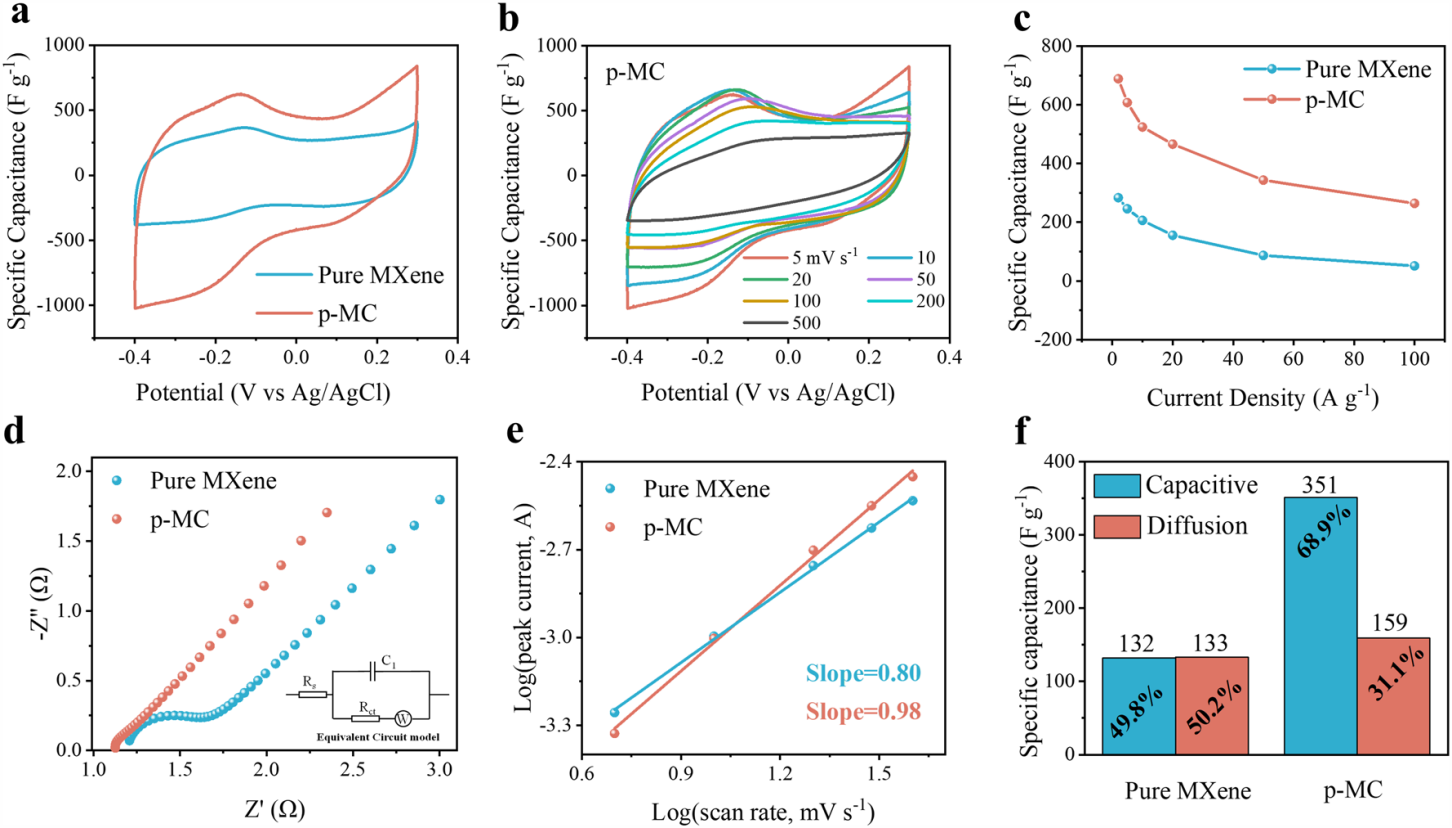
Electrochemical performance of pure MXene and p-MC films. **a** CV curves of pure MXene and p-MC films at 5 mV s^−1^. **b** CV curves of p-MC film from 5 to 500 mV s^−1^. **c** Gravimetric capacitances versus current densities for pure MXene and p-MC films. **d** Nyquist plots of pure MXene and p-MC films. **e** Plots of the log(anodic peak current) versus log(scan rate). **f** Comparison of the capacitance contribution for pure fresh MXene and p-MC films at a scan rate of 5 mV s.^−1^

Owing to the unique structural advantages, p-MC films are expected to exhibit superior electrochemical performance as flexible electrodes for supercapacitors. To assess the electrochemical properties of p-MC film, CV, GCD, and EIS measurements were conducted in three-electrode system in 1 M H_2_SO_4_. To obtain an optimized ratio of CDs in p-MC, the electrochemical properties of fresh and annealed MC films with CDs contents ranging from 0 to 18 mL are shown in Fig. S14. As can be seen, the MC films fabricated with a MXene-to-CDs volume ratio of 10:12 exhibit the highest specific capacitance and capacity retention. Therefore, this MXene-to-CDs volume ratio is used to fabricate the MC and p-MC films in the following discussion. Figure 4a compares the CV profiles of p-MC and pure MXene films in the potential window of −0.4 to 0.3 V (vs Ag/AgCl) at a scan rate of 5 mV s^−1^. The intensity of the redox peaks for p-MC film is much stronger than that of the pure MXene film. Compare to the CV curves of pure MXene (Fig. S15), the CV curves of p-MC film (Fig. 4b) can retain their peak-shapes even at a high scan rate of 500 mV s^−1^ while the peak-shapes for pure MXene have already deformed at 100 mV s^−1^, suggesting that the porous structure and the enlarged interlayer spacing can improve the ion accessibility to pseudocapacitive redox sites on MXene sheets. Figure 4c shows the gravimetric capacitances of the p-MC and pure MXene films. Compare to the pure MXene film (283.7 F g^−1^), the p-MC film delivered a much improved capacitance of 688.9 F g^−1^ at 2 A g^−1^. With the current density increased to 100 A g^−1^, the p-MC film can exhibit a high capacitance retention of 264 F g^−1^, which is more than five times that of pure MXene film (51.4 F g^−1^). Thus, it could be concluded that the ion transport would be more efficient in a porous structure, which leads directly to the improved rate capability for p-MC film (Fig. 4c).

The GCD curves of p-MC and pure MXene electrodes in Fig. S16 are symmetric and nonlinear, which is different from the isosceles-triangular-shaped ideal capacitive behavior, reflecting their intrinsic charge storage mechanisms are the combination of pseudocapacitive and electrolytic double-layer capacitive (EDLC) mechanism. Additionally, the Nyquist plots (Fig. 4d) demonstrate that p-MC film exhibits smaller ohmic resistance (*R*_*s*_) and charge transfer resistance (*R*_*ct*_) than pure MXene (Table S1), revealing the faster charge-transfer kinetics within the p-MC film. The slopes of the straight-line in the Nyquist plots reflect the Warburg impedance of electrodes. The steeper slope of the linear region for p-MC film indicates that the 3D-interconnected porous structure facilitates fast ion diffusion [61].

To shed light on the charge storage mechanism of p-MC electrodes, a power law equation is used to analysis their electrochemical kinetic:

5 $$i=a{v}^{b}$$

where *i* is the peak current, *ν* stands for the scan rate, *a* and *b* are constants. The *b* value can be obtained by fitting the slope of the log (*v*)-log (*i*) plot (Fig. 4e). Generally, *b* = 0.5 indicates a diffusion-controlled process, while *b* = 1 reflects a surface capacitive process [62]. The *b* value of p-MC is determined to be 0.98, implying that the charge storage is mainly governed by the surface capacitive process. The pseudocapacitive contributions to the total capacitance can be further quantified through the following formula (6):

6 $$i(V) ={k}_{1}v+{k}_{2}{v}^{1/2}$$

where *i*(*V*) is the current response, *ν* stands for the scan rate, and *k*_1_*v* and *k*_2_*v*^1/2^ correspond to the surface capacitive and diffusion-controlled process, respectively [63]. The pseudocapacitive contributions of the p-MC and pure MXene films at a scan rate of 5 mV s^−1^ are shown in Fig. 4f. The pseudocapacitive contribution of p-MC is 68.8%, much higher than that of pure MXene (49.8%). Such a high proportion of pseudocapacitive contribution is attributed to the unique structural merits of p-MC film that the intercalation of CDs can help MXene layers expose more internal pseudocapacitive redox sites, leading to improved capacitance and rate performance.

### Flexibility and Application of the p-MC-Based Supercapacitors

**Fig. 5:**
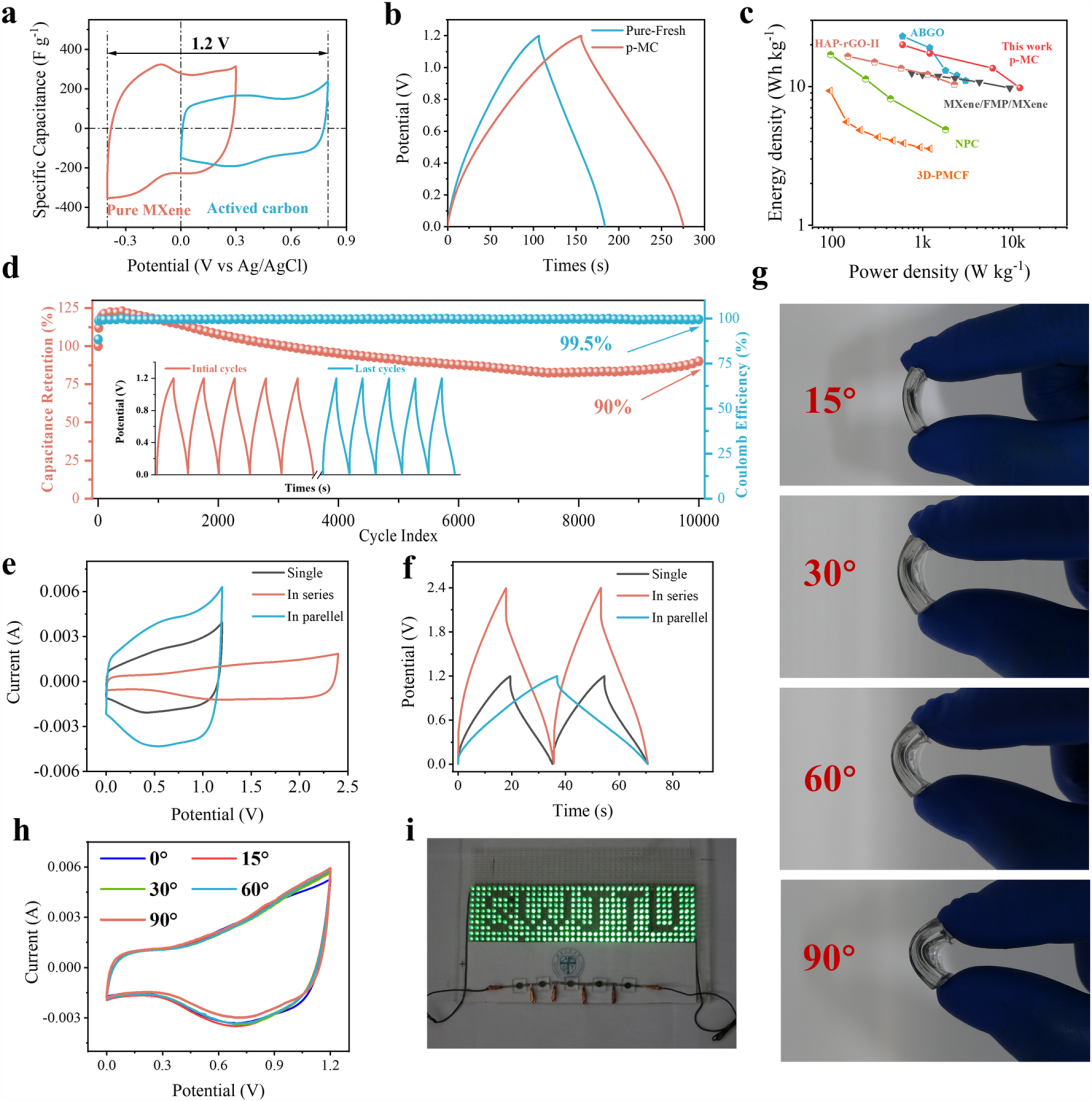
**a** CV curves of pure MXene and AC films at 5 mV s^−1^. **b** GCD curves of pure MXene and p-MC-based asymmetric all-solid-state flexible supercapacitors at a current density of 1 A g^−1^. **c** Ragone plots of p-MC and other MXene-based supercapacitors. **d** Cycling stability performance of p-MC-based supercapacitor at a current density of 8 A g^−1^. **e** CV curves at 10 mV s^−1^, **f** GCD curves at 5 A g^−1^ for single and two p-MC devices connected in series and in parallel. **g** Optical photographs of p-MC-based supercapacitors under various bending angles. **h** CV curves of the p-MC devices bending at various angles. **i** Hundreds of LEDs powered by p-MC-based supercapacitors

To further investigate the practical application potential of p-MC films, the flexible asymmetric all-solid-state supercapacitors were fabricated by taking p-MC film as the negative electrode and AC (active carbon) film as the positive electrode (the detailed assembly process can be found in Experimental Section). As shown in Fig. 5a, the MXene-based electrode and AC electrode exhibit the potential window of -0.4 to 0.3 V and 0 to 0.8 V, respectively, enabling the asymmetric devices to operate at a high potential of 1.2 V. With the scan rate increased from 5 to 50 mV s^−1^ (Fig. S17), a couple of redox peaks were still present on the CV curves of p-MC devices. As calculated from the GCD curves (Figs. 5b and S18–S19), the p-MC-based asymmetric supercapacitor delivers capacitances of 99.8, 86.7, and 68.2 F g^−1^ at 1, 2, and 10 A g^−1^, respectively, which are higher than those of pure MXene-based device. Figure 5c shows the Ragone plots of p-MC-based supercapacitors and many other previously reported MXene-based supercapacitors. The p-MC-based flexible supercapacitors deliver a maximum energy density 20 Wh kg^−1^ at a power density of 600 W kg^−1^, which is higher than that of many reported MXene-based supercapacitors [47, 64–70]. Furthermore, Fig. 5d depicts the long-term cycling stability of p-MC-based supercapacitor, where a high capacitance retention of 90% (with a superior Coulombic efficiency of 99.5%) is maintained after 10,000 cycles, indicating its excellent cycling stability.

To meet the urgent demand for high operating voltage and high-capacity applications, the p-MC-based supercapacitors are integrated together in series and in parallel. As depicted in Fig. 5e, f, two p-MC-based supercapacitors constructed in series afford an operating voltage of 2.4 V, which is twice that of a single device, enabling high energy output (*E* = 1/2 *CV*^2^). When constructing two p-MC-based supercapacitors in parallel, the obtained device can deliver nearly doubled discharge time as well as current output. The solid-state supercapacitors can be bent at various angles (i.e., 0°, 15°, 60°, and 90°), demonstrating their good mechanical flexibility (Fig. 5g). In addition, the CV curves of the p-MC-based supercapacitor under various bending angles (0° to 90°) are almost unchanged (Fig. 5h), suggesting that the device can maintain a stable energy-output during the distorting process. As a demonstration, a panel integrated with about 400 LEDs can be successfully powered by the five-tandem cells (Fig. 5i), showing the great practical application potential of the flexible p-MC-based supercapacitors.

## Conclusions

In summary, a flexible and 3D-interconnected porous MXene/ Carbon Dots (p-MC) composite film was fabricated via a facile thermal annealing process for high-performance flexible supercapacitor. The introducing of CDs enlarges the interlayer spacing of MXene nanosheets, allowing the exposure of more inner pseudocapacitive redox sites and efficient ion diffusion. Comprehensive characterizations reveal that the NH_3_ released from the pyrolysis of CDs etches the MXene nanosheets and creates well-distributed in-plane macropores, thus reducing the ion-path tortuosity in the vertical direction and guaranteeing fast ion transportation. When used as electrode for flexible supercapacitor, p-MC film delivers a high specific capacitance of 688.9 F g^−1^ at 2 A g^−1^ and maintain a high capacitance of 264.3 F g^−1^ even at 100 A g^−1^. Moreover, the p-MC film-based asymmetric flexible supercapacitor exhibits a high energy density (20 Wh Kg^−1^) and superior cycling stability (90% capacitance retention after 10,000 cycles). This work proposes an efficient strategy for constructing porous flexible MXene films and may pave the way to develop high-performance supercapacitors.

### Supplementary Information

Below is the link to the electronic supplementary material.

Supplementary file1 (PDF 1571 kb)

## References

[1] Zhang ZT, Wang WC, Jiang YW, Wang YX, Wu YL (2022). High-brightness all-polymer stretchable LED with charge-trapping dilution. Nature.

[2] Meng XJ, Cai CC, Luo B, Liu T, Shao YZ (2023). Rational design of cellulosic triboelectric materials for self-powered wearable electronics. Nano-Micro Lett..

[3] Zhi CW, Shi S, Zhang S, Si YF, Yang JQ (2023). Bioinspired all-fibrous directional moisture-wicking electronic skins for biomechanical energy harvesting and all-range health sensing. Nano-Micro Lett..

[4] Cheng YF, Xie YM, Liu ZY, Yan SW, Ma YN (2023). Maximizing electron channels enabled by MXene aerogel for high-performance self-healable flexible electronic skin. ACS Nano.

[5] Zhao X, Zhang Z, Liao QL, Xun XC, Gao FF (2020). Self-powered user-interactive electronic skin for programmable touch operation platform. Sci. Adv..

[6] Zhang YF, Xu ZS, Yuan Y, Liu CY, Zhang M (2023). Flexible antiswelling hotothermal-therapy MXene hydrogel-based epidermal sensor for intelligent human–machine interfacing. Adv. Funct. Mater..

[7] Zhang DD, Du JH, Zhang WM, Tong B, Sun Y (2023). Carrier transport regulation of pixel graphene transparent electrodes for active-matrix organic light-emitting diode display. Small.

[8] Kim SK, Lee GH, Jeon C, Han HH, Kim SJ (2022). Bimetallic nanocatalysts immobilized in nanoporous hydrogels for long-term robust continuous glucose monitoring of smart contact lens. Adv. Mater..

[9] Meng KK, Xiao XH, Liu ZH, Shen SH, Tat TJ (2022). Kirigami-inspired pressure sensors for wearable dynamic cardiovascular monitoring. Adv. Mater..

[10] Yang HT, Li JL, Xiao X, Wang JH, Li YF (2022). Topographic design in wearable MXene sensors with in-sensor machine learning for full-body avatar reconstruction. Nat. Commun..

[11] Lin Y, Kang Q, Liu YJ, Zhu YK, Jiang PK (2023). Flexible, highly thermally conductive and electrically insulating phase change materials for advanced thermal management of 5G base stations and thermoelectric generators. Nano-Micro Lett..

[12] Cheng T, Yang XL, Yang S, Li L, Liu ZT (2022). Flexible transparent bifunctional capacitive sensors with superior areal capacitance and sensing capability based on PEDOT:PSS/MXene/Ag grid hybrid electrodes. Adv. Funct. Mater..

[13] Liang J, Tian B, Li SQ, Jiang CZ, Wu W (2020). All-printed MnHCF-MnO_x_-based high-performance flexible supercapacitors. Adv. Energy Mater..

[14] Wang JH, Jiang DG, Du YQ, Zhang MZ, Sun YS (2023). Strong Ti_3_C_2_T_x_ MXene-based composite films fabricated through bioinspired bridging for flexible Energy storage devices. Small.

[15] Xu T, Song Q, Liu K, Liu HY, Pan JJ (2023). Nanocellulose-assisted construction of multifunctional MXene-based aerogels with engineering biomimetic texture for pressure sensor and compressible electrode. Nano-Micro Lett..

[16] Wang D, Zhou CK, Filatov AS, Cho WJ, Lagunas F (2023). Direct synthesis and chemical vapor deposition of 2D carbide and nitride MXenes. Science.

[17] Chae S-U, Yi SH, Yoon J, Hyun JC, Doo S (2022). Highly defective Ti_3_CNT -MXene-based fiber membrane anode for lithium metal batteries. Energy Stor. Mater..

[18] Naguib M, Kurtoglu M, Presser V, Lu J, Niu JJ (2011). Two-dimensional nanocrystals produced by exfoliation of Ti_3_AlC_2_. Adv. Mater..

[19] VahidMohammadi A, Rosen J, Gogotsi Y (2021). The world of two-dimensional carbides and nitrides (MXenes). Science.

[20] Seenivasan S, Shim KI, Lim C, Kavinkumar T, Sivagurunathan AT (2023). Boosting pseudocapacitive behavior of supercapattery electrodes by incorporating a schottky junction for ultrahigh energy density. Nano-Micro Lett..

[21] Xu XY, Zhang ZN, Xiong R, Lu GD, Zhang J (2022). Bending resistance covalent organic framework superlattice:"nano-hourglass"-induced charge accumulation for flexible in-plane micro-supercapacitors. Nano-Micro Lett..

[22] Boota M, Gogotsi Y (2018). MXene—conducting polymer asymmetric pseudocapacitors. Adv. Energy Mater..

[23] Shi B, Li L, Chen AB, Jen TC, Liu XY (2021). Continuous fabrication of Ti_3_C_2_T_x_ MXene-based braided coaxial zinc-ion hybrid supercapacitors with Improved Performance. Nano-Micro Lett..

[24] Wang YM, Wang X, Li XL, Bai Y, Xiao HH (2019). Engineering 3D ion transport channels for flexible MXene films with superior capacitive performance. Adv. Funct. Mater..

[25] Huang HH, Chu X, Xie YT, Zhang BB, Wang ZX (2022). Ti_3_C_2_T_x_ MXene-based micro-supercapacitors with ultrahigh volumetric energy density for all-in-one Si-electronics. ACS Nano.

[26] Wang L, Ma ZL, Qiu H, Zhang YL, Yu Z (2022). Significantly enhanced electromagnetic interference shielding performances of epoxy nanocomposites with long-range aligned lamellar structures. Nano-Micro Lett..

[27] Liu YT, Zhang P, Sun N, Anasori B, Zhu QZ (2018). Self-assembly of transition metal oxide nanostructures on MXene nanosheets for fast and stable lithium storage. Adv. Mater..

[28] Jiao L, Zhang C, Geng CN, Wu SC, Li H (2019). Capture and catalytic conversion of polysulfides by in situ built TiO_2_-MXene heterostructures for lithium–sulfur batteries. Adv. Energy Mater..

[29] Chen C, Xie XQ, Anasori B, Sarycheva A, Makaryan T (2018). MoS_2_ -on-MXene heterostructures as highly reversible anode materials for lithium-ion batteries. Angew. Chem. Int. Ed..

[30] Cai CY, Zhou WB, Fu Y (2021). Bioinspired MXene nacre with mechanical robustness for highly flexible all-solid-state photothermo-supercapacitor. Chem. Eng. J..

[31] Zhu MS, Huang Y, Deng QH, Zhou J, Pei ZG (2016). Highly flexible, freestanding supercapacitor electrode with enhanced performance obtained by hybridizing polypyrrole chains with MXene. Adv. Energy Mater..

[32] Wei SH, Ma JL, Wu DL, Chen B, Du CY (2023). Constructing flexible film electrode with porous layered structure by MXene/SWCNTs/PANI ternary composite for efficient low-grade thermal energy harvest. Adv. Funct. Mater..

[33] Zhang P, Li JP, Yang DY, Soomro RA, Xu B (2022). Flexible carbon dots-intercalated MXene film electrode with outstanding volumetric performance for supercapacitors. Adv. Funct. Mater..

[34] Zhao MQ, Ren CE, Ling Z, Lukatskaya MR, Zhang C (2015). Flexible MXene/carbon nanotube composite paper with high volumetric capacitance. Adv. Mater..

[35] Yue Y, Liu NH, Ma YN, Wang SL, Liu WJ (2018). Highly self-healable 3D microsupercapacitor with MXene-graphene composite aerogel. ACS Nano.

[36] Wang YS, Cui YP, Kong DQ, Wang XN, Li B (2021). Stimulation of surface terminating group by carbon quantum dots for improving pseudocapacitance of Ti3C2Tx MXene based electrode. Carbon.

[37] Lukatskaya MR, Kota S, Lin ZF, Zhao M-Q, Shpigel N (2017). Ultra-high-rate pseudocapacitive energy storage in two-dimensional transition metal carbides. Nat. Energy.

[38] Bao WZ, Tang X, Guo X, Choi SH, Wang CY (2018). Porous cryo-dried MXene for efficient capacitive deionization. Joule.

[39] Bu FX, Zagho MM, Ibrahim Y, Ma B, Elzatahry A (2020). Porous MXenes: synthesis, structures, and applications. Nano Today.

[40] Liu TY, Zhang F, Song Y, Li Y (2017). Revitalizing carbon supercapacitor electrodes with hierarchical porous structures. J. Mater. Chem. A.

[41] Chen NJ, Zhou YH, Zhang SL, Huang HC, Zhang CF (2021). Tailoring Ti_3_CNT_x_ MXene via an acid molecular scissor. Nano Energy.

[42] Sun MY, Qu SN, Hao ZD, Ji WY, Jing PT (2014). Towards efficient solid-state photoluminescence based on carbon-nanodots and starch composites. Nanoscale.

[43] Zhai YC, Wang Y, Li D, Zhou D, Jing PT (2018). Red carbon dots-based phosphors for white light-emitting diodes with color rendering index of 92. J. Colloid Interface Sci..

[44] Chu X, Wang YH, Cai LC, Huang HC, Xu Z (2022). Boosting the energy density of aqueous MXene-based supercapacitor by integrating 3D conducting polymer hydrogel cathode. SusMat.

[45] Wang YB, Chen NJ, Liu Y, Zhou XF, Pu B (2022). MXene/Graphdiyne nanotube composite films for Free-Standing and flexible Solid-State supercapacitor. Chem. Eng. J..

[46] Wang D, Zhou CK, Filatov AS, Cho W, Lagunas F (2023). Direct synthesis and chemical vapor deposition of 2Dcarbide and nitride MXenes. Science.

[47] Yan J, Ren CE, Maleski K, Hatter CB, Anasori B (2017). Flexible MXene/graphene films for ultrafast supercapacitors with outstanding volumetric capacitance. Adv. Funct. Mater..

[48] Yang QY, Xu Z, Fang B, Huang TQ, Cai SY (2017). MXene/graphene hybrid fibers for high performance flexible supercapacitors. J. Mater. Chem. A.

[49] Chen NJ, Huang HC, Xu Z, Xie YT, Xiong D (2020). From high-yield Ti3AlCN ceramics to high-quality Ti_3_CNT_x_ MXenes through eliminating Al segregation. Chin. Chem. Lett..

[50] Luo H, Yang YS, Lu LW, Li GX, Wang XH (2023). Highly-dispersed nano-TiB_2_ derived from the two-dimensional Ti_3_CN MXene for tailoring the kinetics and reversibility of the Li-Mg-B-H hydrogen storage material. Appl. Surf. Sci..

[51] Zhu JW, Wang M, Lyu MQ, Jiao YL, Du AJ (2018). Two-Dimensional titanium carbonitride MXene for High-Performance sodium ion Batteries. ACS Appl. Nano Mater..

[52] Song Y, Wang YB, Zhao YX, Cheng LL, Han GF (2023). Lattice distorted rhodium nanocrystals in porous nanofiber toward aqueous zinc-CO_2_ system. ACS Mater. Lett..

[53] Hantanasirisakul K, Alhabeb M, Lipatov A, Maleski K, Anasori B (2019). Effects of synthesis and processing on optoelectronic properties of titanium carbonitride MXene. Chem. Mater..

[54] Jindata W, Hantanasirisakul K, Eknapakul T, Denlinger JD, Sangphet S (2021). Spectroscopic signature of negative electronic compressibility from the Ti core-level of titanium carbonitride MXene. Appl. Phys. Rev..

[55] Zhang BL, Ju ZJ, Xie QF, Luo JM, Du L (2023). Ti_3_CNT_x_ MXene/rGO scaffolds directing the formation of a robust, layered SEI toward high-rate and long-cycle lithium metal batteries. Energy Stor. Mater..

[56] Ma M, Bai YH, Song XD, Wang JF, Su WG (2020). Investigation into the co-pyrolysis behaviors of cow manure and coal blending by TG-MS. Sci. Total. Environ..

[57] Paulose S, Thomas D, Jayalatha T, Rajeev R, George BK (2016). TG–MS study on the kinetics and mechanism of thermal decomposition of copper ethylamine chromate, a new precursor for copper chromite catalyst. J. Therm. Anal. Calorim..

[58] Strauss V, Wang HZ, Delacroix S, Ledendecker M, Wessig P (2020). Carbon nanodots revised: the thermal citric acid/urea reaction. Chem. Sci..

[59] Tian L, Liu TT, Yang JZ, Yang HY, Liu ZW (2023). Pyrolytic kinetics, reaction mechanisms and gas emissions of waste automotive paint sludge via TG-FTIR and Py-GC/MS. J. Environ. Manage..

[60] Volli V, Varma R, Pradhan D, Panda AK, Singh RK (2023). Thermal degradation behaviour, kinetics, and thermodynamics of Bombax Malabarica seeds through TG-FTIR and Py-GC/MS analysis. Sustain. Energy Tech..

[61] Wang Y, Lu Q, Li F, Guan D, Bu Y (2023). Atomic-scale configuration enables fast hydrogen migration for electrocatalysis of acidic hydrogen evolution. Adv. Funct. Mater..

[62] Brezesinski T, Wang J, Tolbert SH, Dunn B (2010). Ordered mesoporous alpha-MoO_3_-x with iso-oriented nanocrystalline walls for thin-film pseudocapacitors. Nat. Mater..

[63] Kim HS, Cook JB, Lin H, Ko JS, Tolbert SH (2017). Oxygen vacancies enhance pseudocapacitive charge storage properties of MoO_3-x_. Nat. Mater..

[64] Ghosh K, Pumera M (2021). MXene and MoS_3-x_ coated 3D-Printed hybrid electrode for solid-state asymmetric supercapacitor. Small Methods.

[65] Jiang Q, Kurra N, Alhabeb M, Gogotsi Y, Alshareef HN (2018). All pseudocapacitive MXene-RuO_2_ asymmetric supercapacitors. Adv. Energy Mater..

[66] Liu WH, Wang ZQ, Su YL, Li QW, Zhao ZG (2017). Molecularly stacking manganese dioxide/titanium carbide sheets to produce highly flexible and conductive film electrodes with improved pseudocapacitive performances. Adv. Energy Mater..

[67] Pan ZH, Ji XH (2019). Facile synthesis of nitrogen and oxygen co-doped C@Ti_3_C_2_ MXene for high performance symmetric supercapacitors. J. Power. Sour..

[68] Wang X, Li H, Li H, Lin S, Ding W (2020). 2D/2D 1T-MoS_2_/Ti_3_C_2_ MXene heterostructure with excellent supercapacitor performance. Adv. Funct. Mater..

[69] Wang YM, Wang X, Li XF, Li XL, Liu Y (2020). A high-performance, tailorable, wearable, and foldable solid-state supercapacitor enabled by arranging pseudocapacitive groups and MXene flakes on textile electrode surface. Adv. Funct. Mater..

[70] Yang L, Zheng W, Zhang P, Chen J, Zhang W (2019). Freestanding nitrogen-doped d-Ti_3_C_2_/reduced graphene oxide hybrid films for high performance supercapacitors. Electrochim. Acta.

