# Supplementary material for: NH3-Induced In Situ Etching Strategy Derived 3D-Interconnected Porous MXene/Carbon Dots Films for High Performance Flexible Supercapacitors
Source: Nanomicro Lett. 2023 Oct 18;15:231. doi: 10.1007/s40820-023-01204-4 (PMC10584800; doi:10.1007/s40820-023-01204-4)
Supplement: Supplementary file 1 — Supplementary file1 (PDF 1571 kb) [file 40820_2023_1204_MOESM1_ESM.pdf]

Supporting Information for

NH3-Induced In-Situ Etching Strategy Derived 3D-Interconnected Porous MXene/Carbon Dots Films for High Performance Flexible Supercapacitors

Yongbin Wang[1], Ningjun Chen[1], Bin Zhou[3], Xuefeng Zhou[3], Ben Pu[1], Jia Bai[1], Qi Tang[1], Yan Liu[1,*], and Weiqing Yang[1,2,*]

[1]Key Laboratory of Advanced Technologies of Materials, Ministry of Education, School of Materials Science and Engineering, Southwest Jiaotong University, Chengdu 610031, P. R. China

[2]Research Institute of Frontier Science, Southwest Jiaotong University, Chengdu 610031, P. R. China

[3]Sichuan Research Center of New Materials, Institute of Chemical Materials, China Academy of Engineering Physics, Chengdu 610200, P. R. China

*Corresponding authors. E-mail: y_liu@swjtu.edu.cn (Yan Liu), wqyang@swjtu.edu.cn (Weiqing Yang)

Supplementary Figures

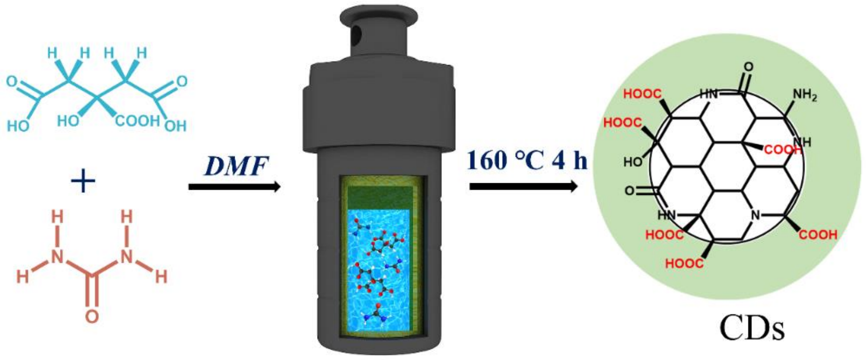

Fig. S1 Schematic illustration of the fabrication process of CDs and the structure of CDs

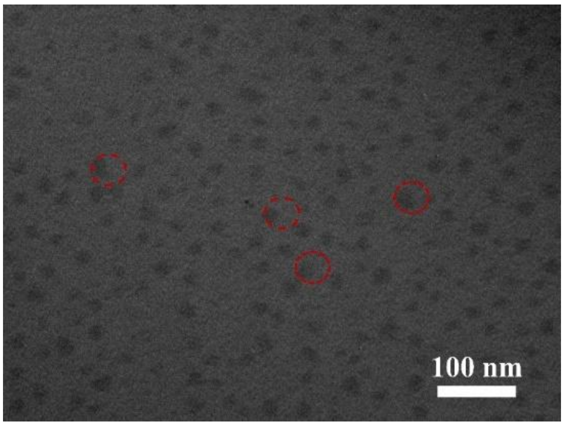

Fig. S2 The TEM image of CDs

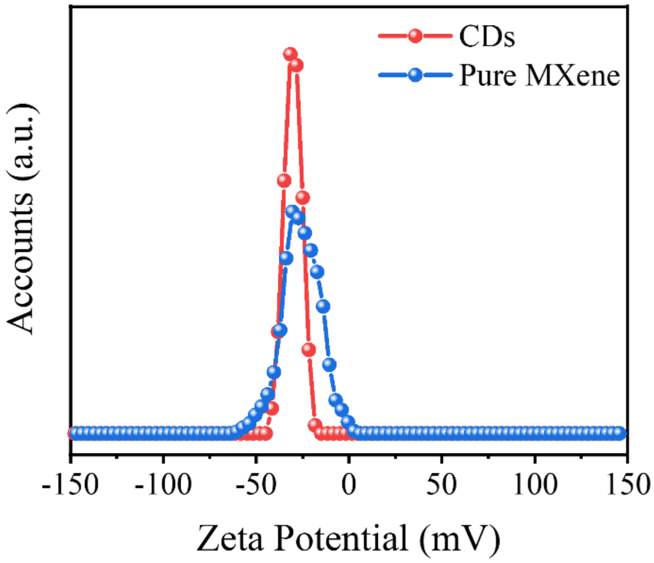

Fig. S3 Zeta potential of pure MXene and CDs

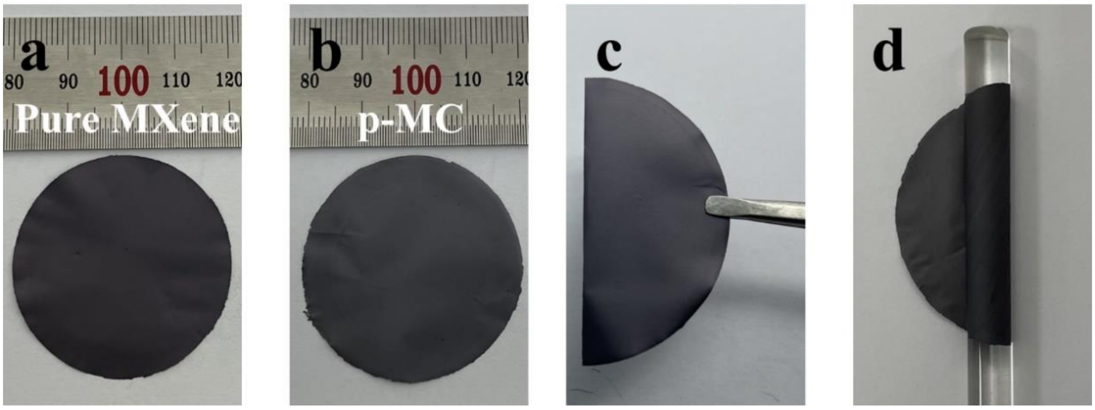

Fig. S4 Digital images of **a** pure MXene and **b** p-MC. **c, d** Fhe flexibility of p-MC

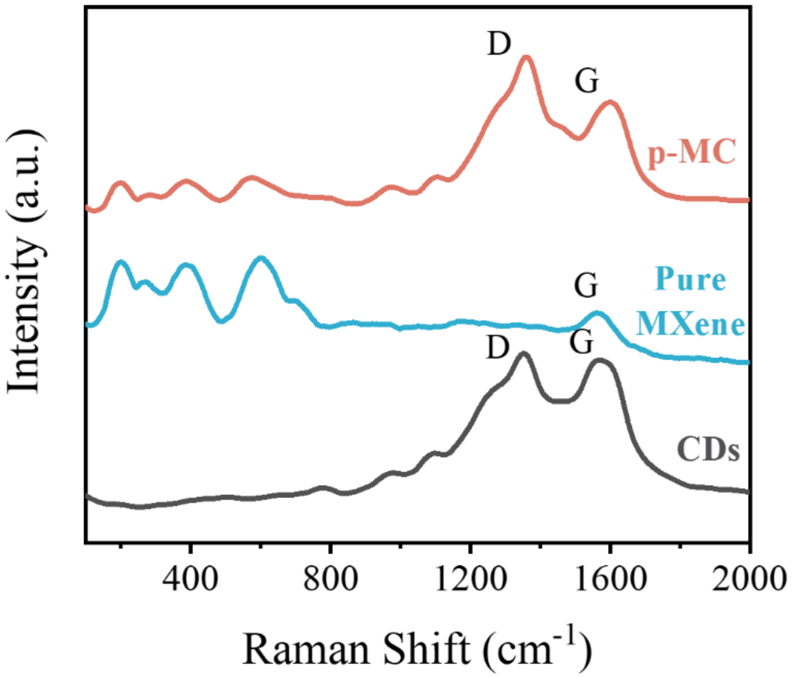

Fig. S5 Raman spectra of CDs, pure MXene, and p-MC

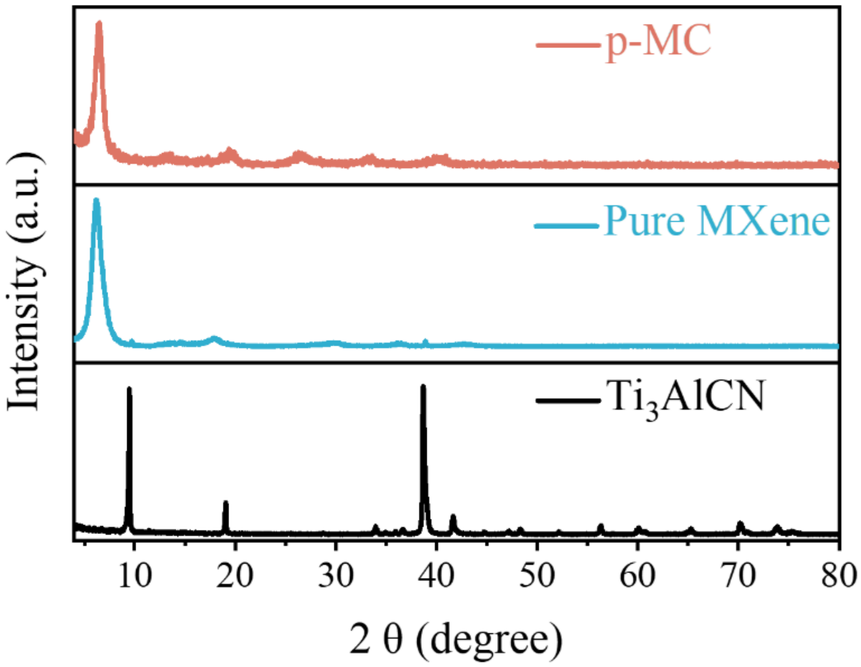

Fig. S6 XRD patterns of Ti_3AlCN, pure MXene, and p-MC

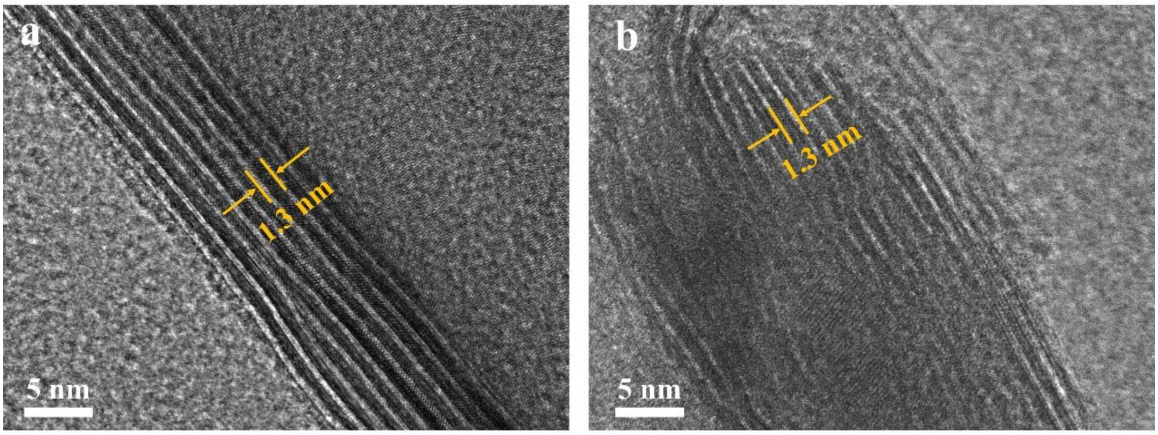

Fig. S7 HRTEM images of **a** pure MXene and **b** p-MC films

Discussion 1. X-ray diffraction (XRD) and HRTEM characterization were carried to investigate the influence of CDs intercalation for the microstructure of MXene. As shown in Fig. S6, the 002 peaks for pure MXene and p-MC films are all located at approximately 6.3°, implying that the interplanar spacing of MXene are very similar in pure MXene and p-MC films. The HRTEM images of the pure MXene and p-MC films also exhibit the similar interplanar spacing of approximately 1.3 nm, which is in accordance with the XRD results. Thus, we can conclude that the intercalation of CDs into the MXene films can enlarge the interlayer spacing between MXene sheets but not changes the interplanar spacing of MXene.

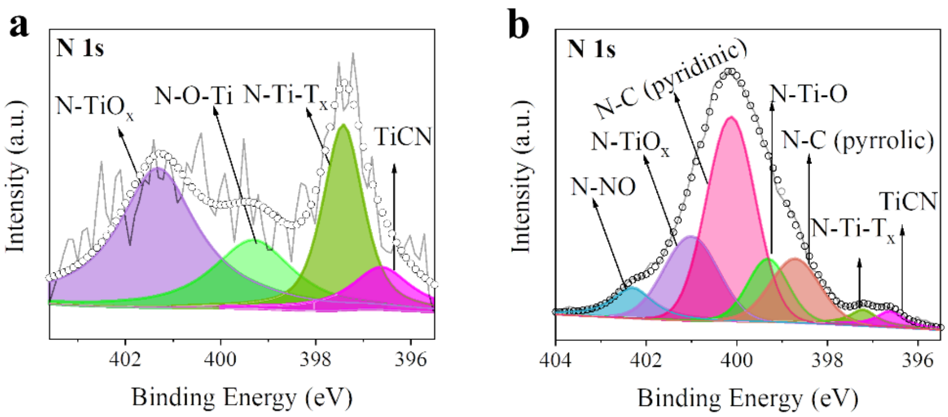

Fig. S8 The N 1s XPS spectra of **a** Pure MXene and **b** p-MC films

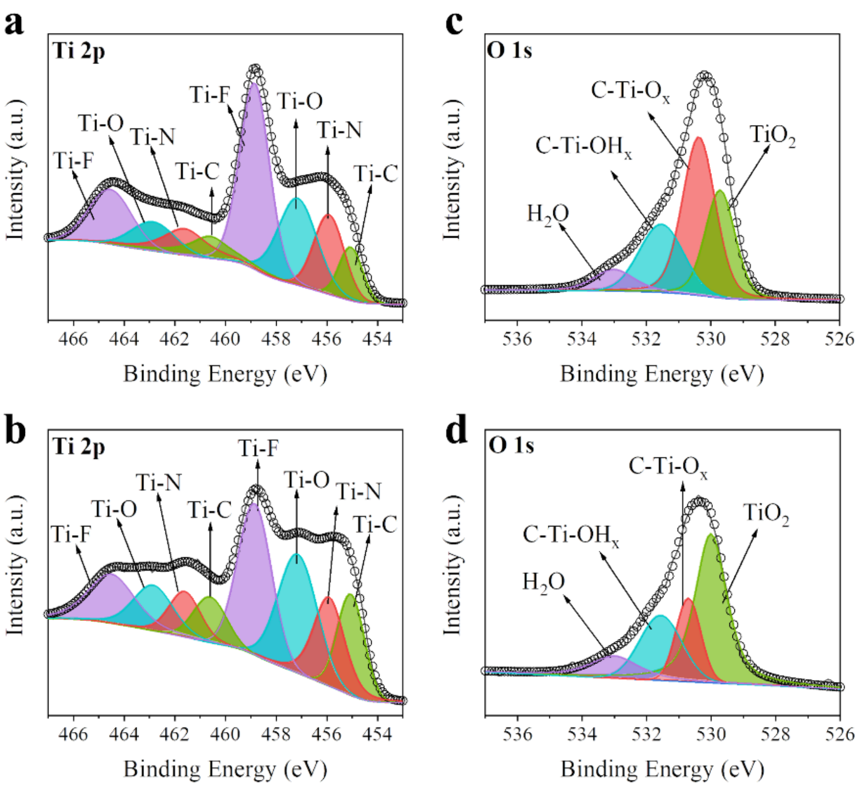

Fig. S9 The Ti 2p XPS spectra for **a** pure MXene and **b** p-MC film. The O 1s, XPS spectra for **c** pure MXene and **d** p-MC film

Discussion 2. The Ti 2p and O 1s XPS spectra were carried to investigate the changes of functional groups on the surface of p-MC (Fig. S9). As the Ti 2p XPS spectra shown in Fig. S9a and S9b, the p-MC film shows weaker peak intensity of Ti-F bond, indicating the decrease of F-containing functional groups on the surface of MXene [S1-S2]. In the O 1s XPS spectra (Fig. S9c, d), the p-MC film also exhibits a weaker C-Ti-O_x peak and a stronger TiO_2 peak than pure MXene, suggesting the annealing treatment can remove part of the O-containing functional groups and cause more oxidation of surface Ti atoms for p-MC film [S3-S6]. Thus, we conclude that the annealing treatment can remove part of O-containing and F-containing functional groups and lead to partial oxidation for Ti atoms on the surface of MXene.

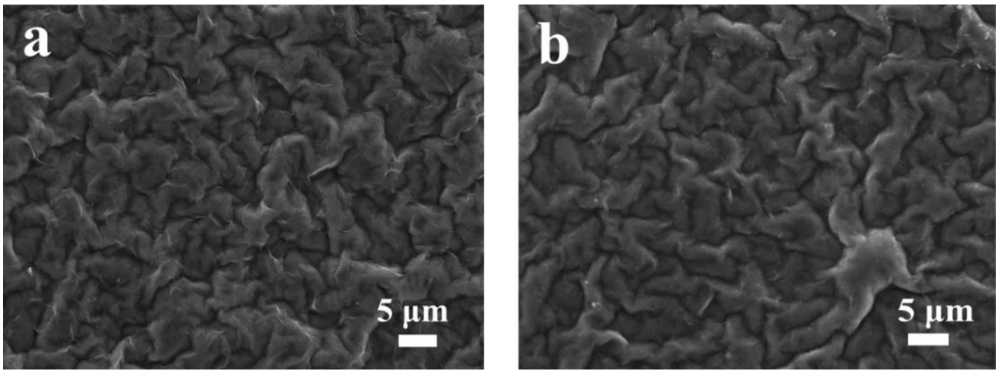

Fig. S10 Top-view SEM images of **a** pure fresh MXene and **b** annealed pure MXene films

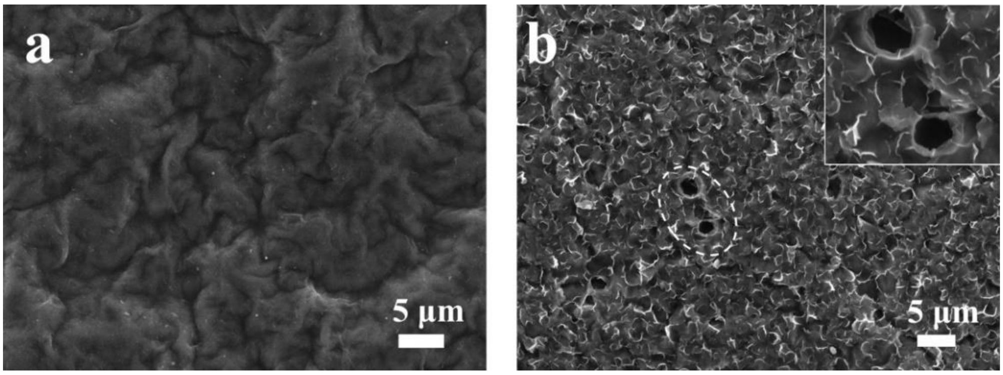

Fig. S11 Top-view SEM images of **a** fresh MC and **b** p-MC films

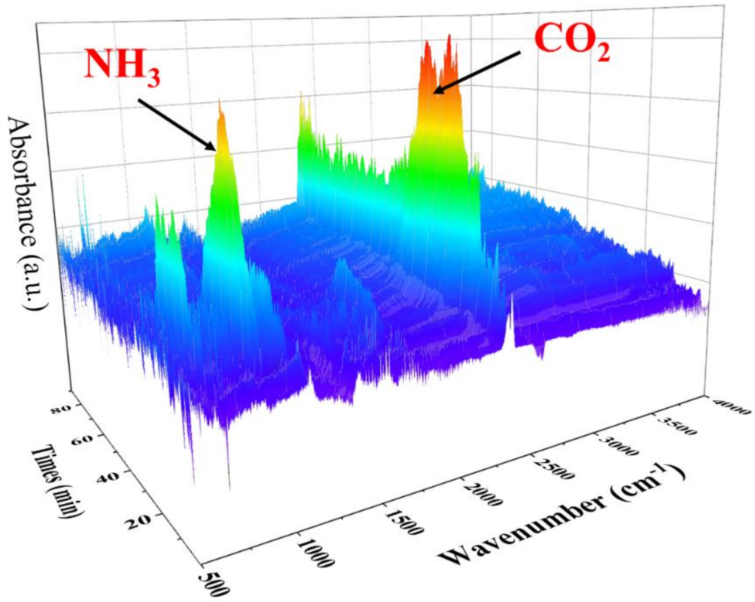

Fig. S12 The 3D infrared spectra for CDs

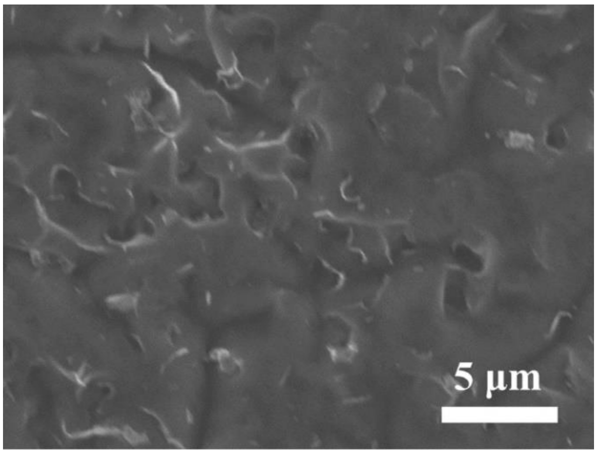

Fig. S13 SEM image of pure MXene annealed at NH_3 atmosphere

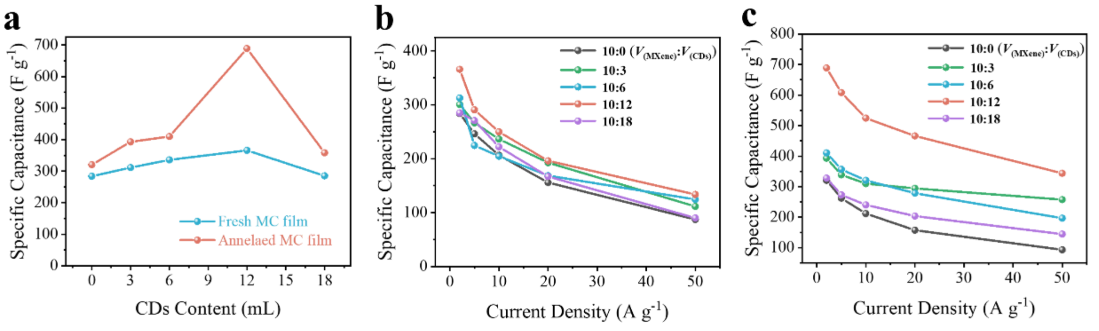

Fig. S14 Electrochemical performance for MC-n films and annealed MC-n films. **a** Capacitance of the fresh and annealed film electrodes as a function of CDs content at 2 A g^{-1}. **b** Rate performance of fresh MC-n films from 2 A g^{-1} to 50 A g^{-1}. **c** Rate performance of annealed MC-n films from 2 A g^{-1} to 50 A g^{-1}

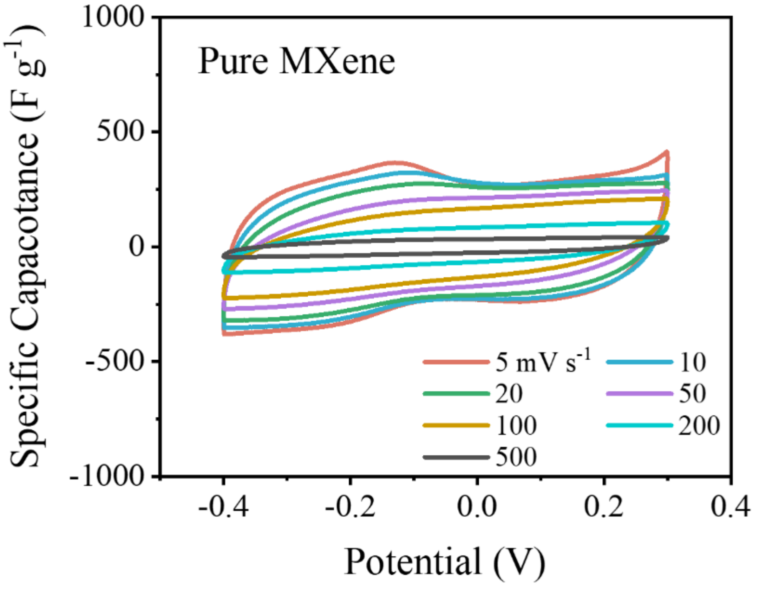

Fig. S15 CV curves of pure MXene film from 5 to 500 mV s^{-1}

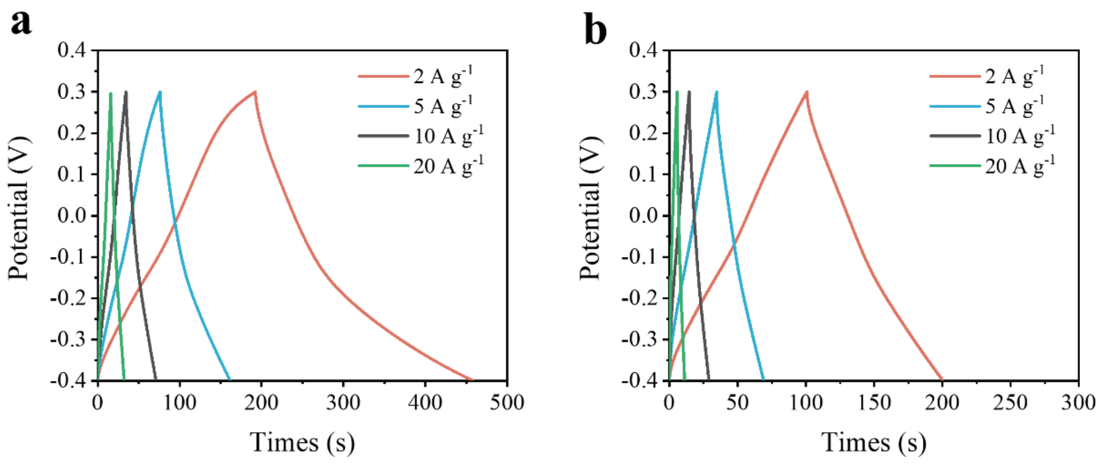

Fig. S16 GCD curves of **a** p-MC and **b** pure MXene films from 2 to 20 A g^{-1}

Table S1 Equivalent circuit model and corresponding fitting values of pure MXene film and p-MC film

Sample	Equivalent circuit model	R_s (Ω)	R_{ct} (Ω)
Pure MXene	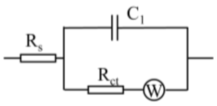	1.202	1.049
p-MC		1.134	0.032

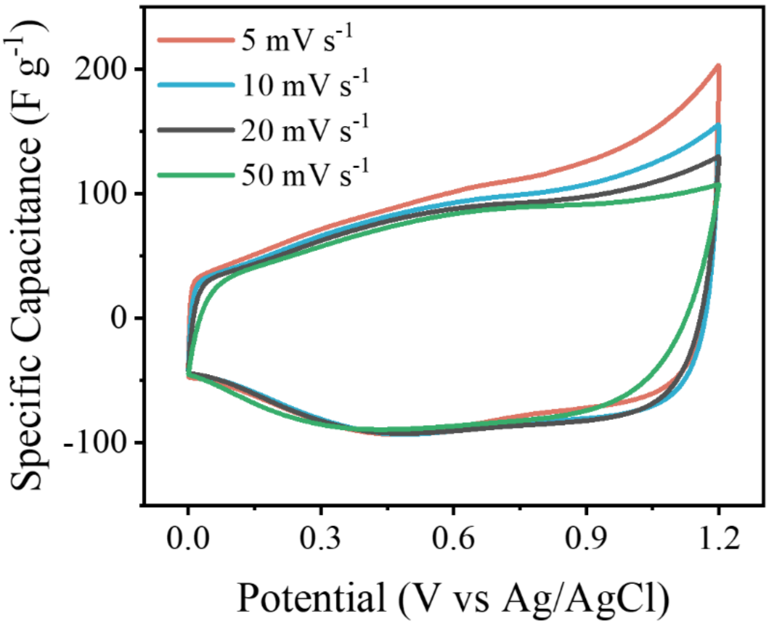

Fig. S17 CV curves of p-MC based flexible solid-state supercapacitor

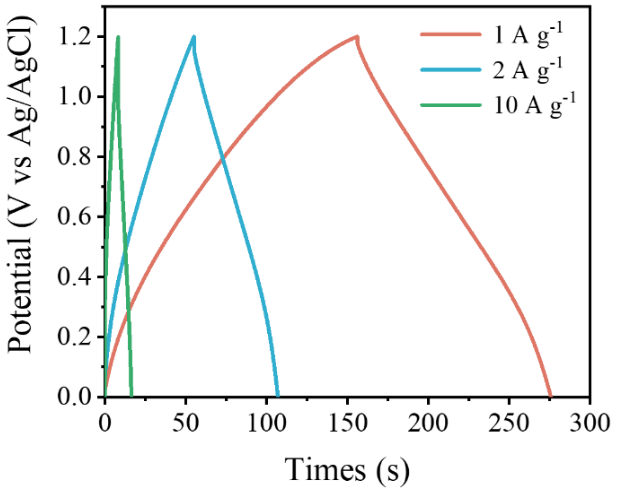

Fig. S18 GCD curves for p-MC based supercapacitor at various current density

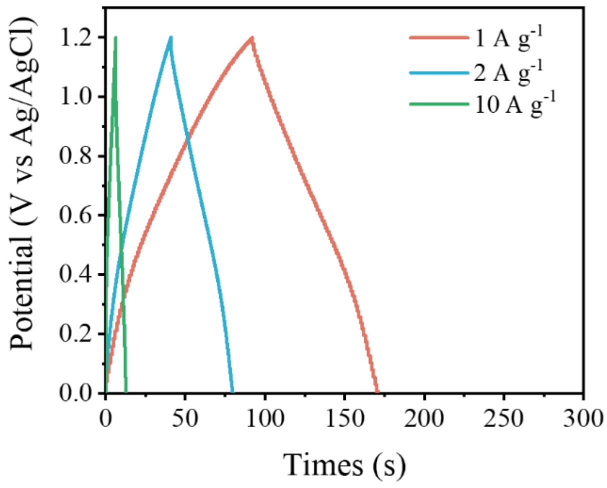

Fig. S19 GCD curves for pure MXene based supercapacitor at various current density

Supplementary References

[S1] J.Z. Jiang, F.Y. Li, S.S. Bai, Y.J. Wang, K. Xiang et al., Carbonitride MXene $Ti_3CN(OH)x$@MoS_2 hybrids as efficient electrocatalyst for enhanced hydrogen evolution. Nano Res. **16**, 4656-4663 (2022). https://doi.org/10.1007/s12274-022-5112-x

[S2] J.W. Zhu, M. Wang, M.Q. Lyu, Y.L. Jiao, A.J. Du et al., Two-Dimensional titanium carbonitride MXene for high-performance sodium ion batteries. ACS Appl. Nano Mater. **1**, 6854-6863 (2018). https://doi.org/10.1021/acsanm.8b01330

[S3] K.Y. Zhang, M.Y. Di, L. Fu, Y. Deng, Y.W. Du et al., Enhancing the magnetism of 2D carbide MXene $Ti_3C_2T_x$ by H_2 annealing. Carbon **157**, 90-96 (2020). https://doi.org/10.1016/j.carbon.2019.10.016

[S4] S.L. Zhang, H.J. Ying, P.F. Huang, J.L. Wang, Z. Zhang et al., Rational design of pillared SnS/$Ti_3C_2T_x$ MXene for superior lithium-ion storage. ACS Nano **14**, 17665-17674 (2020). https://doi.org/10.1021/acsnano.0c08770

[S5] K. Hantanasirisakul, M. Alhabeb, A. Lipatov, K. Maleski, B. Anasori et al., Effects of synthesis and processing on optoelectronic properties of titanium carbonitride MXene. Chem. Mater. **31**, 2941-2951 (2019). https://doi.org/10.1021/acs.chemmater.9b00401

[S6] J. Halim, K.M. Cook, M. Naguib, P. Eklund, Y. Gogotsi et al., X-ray photoelectron spectroscopy of select multi-layered transition metal carbides (MXenes). Appl. Surf. Sci. **362**, 406-417 (2016). https://doi.org/10.1016/j.apsusc.2015.11.089
